# Polyclonal anti-whole cell IgY passive immunotherapy shields against *P. aeruginosa*-induced acute pneumonia and burn wound infections in murine models

**DOI:** 10.1038/s41598-023-50859-x

**Published:** 2024-01-03

**Authors:** Tooba Sadat Ahmadi, Bahador Behrouz, Seyed Latif Mousavi Gargari

**Affiliations:** Department of Biology, Faculty of Basic Sciences, Shahed University, Tehran-Qom Express Way, Tehran, 3319118651 Iran

**Keywords:** Biotechnology, Immunology, Microbiology

## Abstract

*Pseudomonas aeruginosa* (PA) is a multidrug-resistant (MDR) opportunistic pathogen causing severe hospital-, and community-acquired infections worldwide. Thus, the development of effective immunotherapy-based treatments is essential to combat the MDR-PA infections. In the current study, we evaluated the protective efficacy of polyclonal avian antibodies raised against inactivated whole cells of the PAO1 strain in murine models of acute pneumonia and burn wound. The efficacy of generated antibodies was evaluated against different PA strains through several in vitro, ex vivo and in vivo experiments. The results showed that the anti-PAO1-IgY effectively reduced the motility, biofilm formation and cell internalization ability, and enhanced the opsonophagocytic killing of PA strains through the formation of immobilized bacteria and induction of increased cell surface hydrophobicity. Furthermore, immunotherapy with anti-PAO1-IgY completely protected mice against all PA strains in both acute pneumonia and burn wound murine models. It was found to reduce the bacterial loads in infected burned mice through interfering with virulence factors that play vital roles in the early stages of PA infection, such as colonization and cell internalization. The immunotherapy with anti-PAO1-IgYs could be instrumental in developing effective therapies aimed at reducing the morbidity and mortality associated with PA infections.

## Introduction

*Pseudomonas aeruginosa* (PA) is one of the leading healthcare-associated pathogens posing substantial therapeutic challenges in clinical settings. This opportunistic pathogen is responsible for a major proportion of morbidity and mortality in vulnerable immunocompromised individuals with trauma, cystic fibrosis (CF), cancer and burns^1^. The minimal nutritional requirements, great adaptability to adverse environmental conditions and ubiquitous genome-based resistance mechanisms allow this audacious bacterium to cause several hospital-acquired diseases such as pneumonia, bacteremia, sepsis and other life-threatening infections across the globe^2^. *P. aeruginosa* is one of the three top-listed critical antibiotic-resistant pathogens prioritized by the World Health Organization (WHO) requiring urgent inquiry toward expanding alternative therapeutic approaches^3^. In the recent COVID-19 pandemic, *P. aeruginosa* was identified as the second most frequent bacterial co-infection in patients, exacerbating the disease complexity and imposing high expenditures on healthcare infrastructure^4^.

The *P. aeruginosa* pathogenicity arises from its assorted virulence factors and intrinsic, acquired, and adaptive resistance mechanisms. The diverse secreted compounds, surface structural components and quorum sensing network are the major contributors to bacterial competency to cause infections in hosts^5^. Antibiotic therapy has proven inadequate in managing the overwhelming disease burden caused by multidrug-resistant (MDR) PA strains^6^. Despite the vast attempts made over the past few decades to characterize the protective aspects of several empirical and targeted antigens, no vaccine has obtained market authorization yet^7^. While several experimental anti-PA vaccines have undergone preclinical trials, only a handful have advanced to the clinical phases. These vaccine candidates include lipopolysaccharides, surface polysaccharides, outer membrane proteins (OMP), flagella, pili, several recombinant proteins, as well as killed, inactivated or live-attenuated whole-cell preparations^7^. Considering the complex nature and the current knowledge deficiency in dissecting the diverse molecular and mechanistic details of the bacterium, the latter candidate may indeed provide a holistic approach by involving an arsenal of several potentially protective antigens and immunostimulatory components.

Despite promising progress toward developing novel antimicrobials, inactivated whole-cell vaccines (WCV) have yet to be widely applied as an efficacious complement to existing strategies to address public health needs^8^. However, active immunization using a WCV may potentially cause irreparable harm to patients with certain medical conditions, or may be interfered with an underlying immune compromise. Therefore, careful consideration and evaluation of individual patient risk factors and medical history is necessary before deciding on the use of a WCV^8^. As an ongoing quest for developing efficacious anti-PA therapeutics, passive immunotherapy has the advantage of inducing immunity against a wide array of antigens while avoiding the possible disadvantages of active immunization approaches using whole-cell preparations. Egg yolk immunoglobulins are an inexpensive and convenient source of polyclonal antibodies that have been widely used for diagnostic, prophylactic and therapeutic applications^9^.

IgYs have several advantages over other immunoglobulins. IgY has found applications across a range of uses, such as immunodiagnostics, immunotherapy, and affinity purification^9^. Its unique properties render it a preferable choice in cases where conventional IgG antibodies might not deliver the best results^10^. Conversely, as IgY originates from avian species, it proves particularly valuable in research involving mammals^10^. When employed as a probe or reagent, IgY minimizes the risk of cross-reactivity with mammalian IgGs, thus diminishing background interference^9^. The distressful and laborious prerequisites for antibody extraction in mammals are substituted by simple egg collection for avian antibody production^9^. IgY possesses a larger structural size compared to IgG, which can confer advantages in certain assays and applications where larger binding molecules are preferred^11^. The utilization of chickens for antibody production also represents higher yields, economic benefits and refined animal welfare^9^. The distinct Fc region structure of IgY, in contrast to IgG, does not trigger activation of the complement system^12^, and exhibits no cross-reactivity with rheumatoid factors^13^ or human anti-mouse antibodies (HAMA,^14^), enhancing the efficacy of human therapeutic platforms by avoiding unwanted interferences^9^.

Two separate clinical trials sponsored by Immunsystem AB^15^ and Mukoviszidose Institut gGmbH^7^ were underway to evaluate the safety and efficacy of polyclonal avian antibodies raised against formaldehyde-fixed whole-cell PAO1 strain in addition to other PA strains, administered as a daily mouthwash to prevent infection acquisition in CF patients. To our knowledge, there have been no reported instances of anti-PA IgY antibodies being examined against acute pneumonia or burn wound models. Thus, the current study aimed to assess the protective efficacy of polyclonal anti-single whole-cell PAO1 IgY against PA infections induced by standard and hospital strains in murine models.

## Materials and methods

### Bacterial strains

Avian antibodies were produced using *P. aeruginosa* PAO1 (ATCC 15692) as the antigen. In all experimental assays, heterologous strains of *P. aeruginosa* PAK (ATCC 25102) and a previously characterized MDR hospital strain^16^ (R5) were utilized. The PAK and PAO1 standard strains were provided by the Central Medical Laboratory Services of the Burn Research Center (Tehran, Iran), and the R5 strain was kindly gifted by the Faculty of Pharmacy, Tehran University of Medical Sciences (Tehran, Iran).

### Animal experiments

Eighteen-week-old laying White Leghorn hens were procured from an industrial aviculture for the production of avian antibodies. Female BALB/c mice aged six to eight weeks (Royan Institute, Tehran, Iran) were utilized for in vivo assays.

### Whole-cell inactivation

The PAO1 strain was grown in fresh LB media to determine the colony-forming units (CFU)/mL of the suspension against the optical density (OD) at 600 nm using Plate Count Agar (PCA) method. The inactivated whole-cell (IWC) antigenic mixture was prepared by culturing the bacterial strain in LB broth to an OD_600_ of 0.8. The bacterial pellet was extensively washed and resuspended in phosphate-buffered saline (PBS), followed by an inactivation process through 24-h exposure to 1% (v/v) formalin at 4 °C. Formalin was removed via centrifugation at 10,000 rounds per minute (RPM) for 30 min at 4 °C^17^. The integrity of the inactivation process was confirmed through plating the final IWC on rich media, and monitored for 1 week.

### Hen immunization

The IWC was adjusted to contain 5** × **10^9^ CFUs. The hens were randomly divided into two groups (n = 2), and immunized intramuscularly with the IWC strain at weeks 0, 2 and 5. Two adjuvants were used in combination to induce a high and robust antibody response while minimizing potential adverse side effects^18^; Montanide ISA70VG, utilized at an adjuvant/antigen ratio of 70:30 (v/v), and cytosine-phosphate-guanosine oligodeoxynucleotide (CpG-ODN 2135, sourced from Microsynth, Balgach, Switzerland), administered at quantities of 30, 25, and 10 μg in the first, second and third injections, respectively. The control group received PBS instead of the IWC. Eggs were collected on a daily basis and stored at 4 °C until further experimentation.

### IgY purification

In order to monitor the immunization process, eggs laid at different time points during the injections were used for ELISA assays, and based on obtained results, all other assays were conducted using IgYs extracted from eggs laid at least one week after the last injection. Polyclonal IgYs were purified using a cost-effective procedure as previously described^19^. Eight-fold diluted egg yolks in distilled water were adjusted to pH 5. The solution was then frozen and melted over filtration paper. The IgYs were salted out using 8.8% NaCl at pH 4 for 2 h with agitation. The resulting cloudy solution was centrifuged at 3500×*g* for 20 min at 4 °C. The final sediment was dissolved in sterile PBS. The purity of the avian antibodies was examined using 9% SDS-PAGE (Bio-Rad, USA). The IgY solutions were then lyophilized and stored at room temperature (RT) until further use. The antibody powders were reconstituted to twice the highest concentration investigated in each experiment before use and were then sterilized using a 0.22 mm pore-size membrane filter to avoid bacterial and fungal contamination.

### Immunoreactivity evaluation of IgYs

Indirect enzyme-linked immunosorbent assay (ELISA) was performed to evaluate the immunoreactivity and production kinetics of generated IgYs. Ninety-six-well microplates (Nunc, USA) were coated separately with 10^8^ CFUs of each standard strain in 0.03 M carbonate bicarbonate coating buffer (pH 9.6), and incubated overnight at 4 °C. Wells were then blocked with 0.05% PBS-Tween 20 (PBST) + 5% skimmed milk for 45 min at 37 °C. Five micrograms of each IgY sample were added to each well and plates were incubated for 2 h at 37 °C. The plates were washed five times with PBST and then incubated with horseradish peroxidase (HRP)-conjugated rabbit anti-IgY antibody (A9046, Sigma-Aldrich, 1:10,000-diluted) for 1 h at 37 °C. Tetramethylbenzidine (TMB, Sigma-Aldrich) was added as an HRP substrate for 15 min at RT. The reaction was stopped by adding 3 M H_2_SO_4_ and OD_450_ was recorded using a microplate absorbance reader (Sunrise Absorbance Reader, Tecan). All washings were performed using PBST^20^.

### In vitro experiments

#### Growth inhibition assay

The growth inhibition assay was conducted following the procedure described by Lee et al.^21^ with slight modifications. Various concentrations (ranging from 0.1 to 10 mg/mL) of anti-PAO1-IgY in fresh LB broth were incubated with 2 × 10^7^ CFUs/mL of bacteria of mid-logarithmic phase in sterile test tubes for 6 h at 37 °C with shaking at 250 rpm. One-hundred-microliter aliquots were then serially diluted and plated on LB agar. Plates were incubated at 37 °C overnight, and colonies were counted to determine the CFU/mL of each sample. The control IgY (C-IgY), used at the highest examined concentration (10 mg/mL), and samples without IgY were used as negative and blank (PBS) controls, respectively. Also, ceftazidime dissolved in LB medium at a concentration of 32 μg/mL was used as positive control.

#### Motility inhibition assay

Bacterial strains were incubated in fresh LB broth to reach an OD_600_ of 0.2. Bacterial suspensions were centrifuged and the pellets were washed twice and resuspended in PBS. One-hundred microliter aliquots of suspensions were incubated with different anti-PAO1-IgY concentrations (0.005–10 mg/mL) in a total volume of 500 μL for 1 h at RT with shaking at 130 rpm. Then, 10 μL aliquots were placed on the center of semi-solid LB media with 0.3% (w/v) agar, and incubated at 30 °C for 18 h. Bacterial motility was determined by subtracting the primary diameter from the final diagonal of the growth area. Samples with no IgY were considered as negative control^22^.

#### Bacterial hydrophobicity assay

The antibody-imposed cell surface hydrophobicity (CSH) changes in the bacteria were evaluated through microbial adhesion to hydrocarbon (MATH) assay as previously described^23^ with slight modifications^24^. Fresh mid-logarithmic phase bacteria were washed and resuspended in phosphate urea magnesium sulfate buffer (PUM, 22.2 g/ L K_2_HPO_4_ × 3H_2_O, 7.26 g/L KH_2_PO_4_, 1.8 g/L urea, 0.2 g/L MgSO_4_ × 7H_2_O; pH 7.1) to reach an OD_600_ of 0.63. Four milliliter aliquots were incubated with different test and control IgY concentrations (0.02–2 mg/mL) for 1 h at RT. Three-milliliter aliquots of the IgY-treated bacteria were transferred to separate sterile test tubes and isooctane (0.5 mL) was steadily layered on top. Tubes were vortexed for one minute and the OD_600_ of the aqueous phase was measured after complete phase separation. The hydrophobicity index (HI) was calculated as follows: HI = (initial absorbance–absorbance after phase separation)/(initial absorbance). Samples with no IgYs (PBS) were considered as negative control.

#### Biofilm inhibition assay

Bacterial strains (10^7^ CFUs/well) in fresh glucose-supplemented LB medium (tryptone 10 g/L, yeast extract 5 g/L, NaCl 5 g/L, and glucose 2 g/L) were incubated with different test and control IgY concentrations (0.02–2 mg/mL) in wells of a tissue culture plate overnight at 37 °C under static conditions. The formed biofilms were fixed and stained by absolute methanol and 0.1% crystal violet for 15 and 5 min at RT, respectively. Stained biofilms were incubated with 95% ethanol at 37 °C for 30 min after washing the excess stain. Finally, the OD_575_ was measured using a microplate spectrophotometer. The wells containing only medium were considered as medium control and their mean OD value was subtracted from all other values. Also, wells with no IgY (PBS) were used as positive control^25^.

### Ex vivo experiments

#### Cell internalization assay

Fresh bacterial strains (2 × 10^8^ CFUs/well) were treated with different test and control IgY concentrations (0.05–1 mg/mL) for 1 h at RT. The mixture was resuspended in FBS-free Dulbecco’s Modified Eagle Medium (DMEM) in each well of a tissue culture plate pre-coated with a confluent monolayer of A549 lung epithelial cells (2 × 10^5^ cells/well) and incubated for 90 min at 37 °C. Wells were washed and cells were treated with 100 μg/mL gentamicin for 1 h to eliminate non-internalized bacteria. The A549 cells were then lysed through a 5-min exposure to 0.25% Triton X-100, and the released bacteria were serially diluted and plated onto LB agar. Wells lacking A549 lung epithelial cells, and wells with no IgY were regarded as negative and positive (PBS) controls, respectively^26^.

#### Opsonophagocytic assay

Neutrophils were obtained from healthy human volunteers using a previously described method^27^. In brief, whole blood was steadily layered over the Ficoll density gradient medium in a sterile 50-mL Falcon tube. The neutrophil layer was collected via centrifugation and residual erythrocytes were lysed. Pretreated bacterial strains at different anti-PAO1-IgY concentrations (0.02–1 mg/mL) were incubated with 10^5^ neutrophils with a multiplicity of infection (MOI) of one for 90 min at 37 °C. Hanks balanced salt solution (HBSS) was used as resuspension buffer for all reaction reagents. The surviving bacteria were serially diluted and plated on LB agar. Samples with no IgYs were considered as negative control. The IgY-induced PMN-mediated opsonophagocytic activity was reported as survival percent, calculated as follows: Survival percent (%) = (bacterial CFUs of IgY-pretreated samples at 90 min/bacterial CFUs of samples with no IgY at 90 min) × 100.

### In vivo experiments

#### Burn wound infection

A murine model was employed to assess the protective efficacy of anti-PAO1-IgYs against burn wound infection as previously described^24^. Mice were anesthetized intraperitoneally (IP) with a combination of ketamine (80 mg/kg) and xylazine (12 mg/kg). Then, a third-degree burn wound covering 12–15% of the total body surface area (TBSA) was generated on the shaved backs of mice by applying a 105 °C iron-alloyed probe for 8 s. Half a milliliter of sterile saline was IP administered immediately to avoid hypovolemic shock, and acetaminophen (0.25 mg/mL) was used as post-burn analgesic^16^. To determine the lethal doses (LD) of bacterial strains, the burned site was infected through subscar injection of different doses of bacteria one hour after burn induction, and the survival rates were monitored for one week. Furthermore, considering the outcomes of our prior in vitro and ex vivo experiments and the observed threshold ranges, a pilot study was conducted to ascertain the optimal anti-PAO1-IgY dosage.

Once the optimal anti-PAO1-IgY dose was determined, a subsequent primary experiment was conducted to evaluate the protective efficacy of the prescribed amount via subscar (SC), intravenous (IV) and intraperitoneal routes. One hour after burn induction, mice (n = 6) were infected through subscar injection of a LD of PAO1 strain thoroughly mixed with 1 mg of anti-PAO1-IgY immediately before injection. Mice received the initial treatment dose of IgY (1 mg IgY/100 μL) 2 h thereafter, followed by the same doses at 24, 48, and 72 h post-infection through different administration routes. The control group received PBS instead of IgY, and the survival rates were monitored for one week. Eventually, the intravenous route was selected as the most effective administration route.

The protective efficacy of IgYs was examined as follows; The burned back of mice (n = 8) were infected through subscar injection of the LDs of bacterial strains which were thoroughly mixed with 1 mg of test and control IgYs immediately before injection. The first treatment dose (1 mg IgY/100 μL) was injected intravenously (IV) via the tail vein 2 h thereafter, followed by the same doses at 24, 48, and 72 h post-infection. The PBS-treated mice and burned mice without infection were considered as negative control and burn control groups, respectively. The survival rates were monitored daily for seven days. The LDs for laboratory (PAK and PAO1) and hospital (R5) strains were obtained after 24 and 48 h, respectively. Thus, the bacterial burden examinations were performed at the same time. Three mice from each group were sacrificed through anesthetic overdose injection at 24 and 48 h post-infection to assess the bacterial loads. Blood, liver, spleen and skin biopsy samples were thoroughly homogenized and plated on NA after serial dilution in PBS.

#### Acute pneumonia

Mice were intranasally challenged with lethal doses of PA strains (2 × 10^7^ CFUs) freshly mixed with 1 mg of IgYs directly into one nostril^20^. Mice received two half-doses (500 µg) 2 and 24 h after infection, and were closely monitored for 1 week.

### Statistical analysis

Statistical analyses were performed using SPSS 25.0 software (SPSS Inc., Chicago, Illinois, USA) at a significance level of P ≤ 0.05. All data followed a normal distribution and thus, parametric methods were applied for statistical analysis. One-way ANOVA and Duncan’s multiple range tests were employed to assess the significance of differences among different treatments at each IgY concentration for each strain, as well as between different IgY concentrations for each strain (Supplementary data). Two-way ANOVA and Tukey's multiple comparisons test were used to analyze ELISA and bacterial load examination results. Survival data were analyzed through the Mantel-Cox log-rank test. Results were expressed as mean ± standard error of mean (SEM). GraphPad Prism 8 software was used to plot all graphs.

### Ethics approval

All methods and experiments were performed in accordance with relevant guidelines and regulations, and all experimental protocols were approved by Shahed University Animal Safety Committee’s Guide for the Care and Use of Laboratory Animals (approved code: IR.SHAHED.REC.1395.002). We confirm that this study adheres to the ARRIVE (Animal Research: Reporting of In Vivo Experiments) guidelines for reporting animal research. The Ethics Committee of Shahed University meticulously reviewed and approved our study, ensuring compliance with all relevant ethical guidelines. In accordance with the guidelines set forth by the committee, informed consent was deemed unnecessary and was consequently waived. The blood samples crucial to our study were generously and voluntarily donated by the investigators involved in the research, falling under the category exempt from the requirement for informed consent by the Ethics Committee of Shahed University, relieving us of the requirement for written informed consent from the donors.

## Results

### Purity and immunoreactivity of IgYs

**Figure 1:**
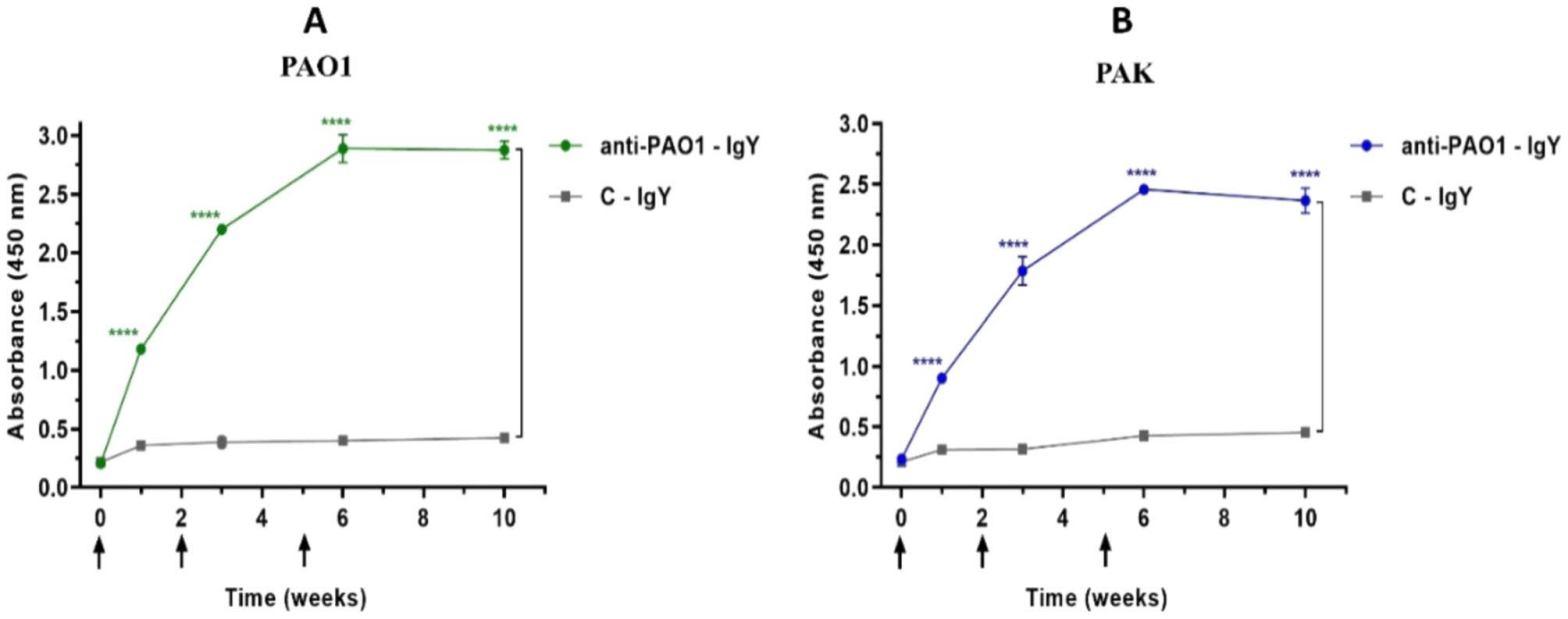
The immunoreactivity of anti-PAO1-IgY; mean comparison of anti-PAO1-IgY reactivity against PAO1 **(A)** and PAK **(B)** strains in comparison to C-IgY at each time point, in terms of absorbance (450 nm), analyzed using Tukey’s multiple comparisons test. Arrows indicate the immunization weeks (0, 2 and 5). Values represent the mean of three independent triplicate experiments ± standard error of mean (SEM), ****P ≤ 0.0001.

IgY antibodies were extracted from egg yolks with an approximate yield of 10–12 mg/mL. SDS-PAGE analysis of purified IgYs revealed two main bands of ~ 65 and ~ 25 kDa, corresponding to heavy and light chains of the antibodies, respectively (Supplementary data, Fig. S1, A). The anti-PAO1-IgY showed significantly higher titers against standard strains in comparison to C-IgY since the first injection (Fig. 1). Although the anti-PAO1-IgY showed a higher immunoreactivity against its homologous strain, a strong response was also observed against the PAK strain. The findings demonstrated a robust immune response in anti-PAO1-IgY group against both strains for a duration of up to 14 weeks following the last injection, as depicted in Supplementary data, Fig. S1B, C.

### The anti-PAO1-IgY reduced the bacterial proliferation

**Figure 2:**
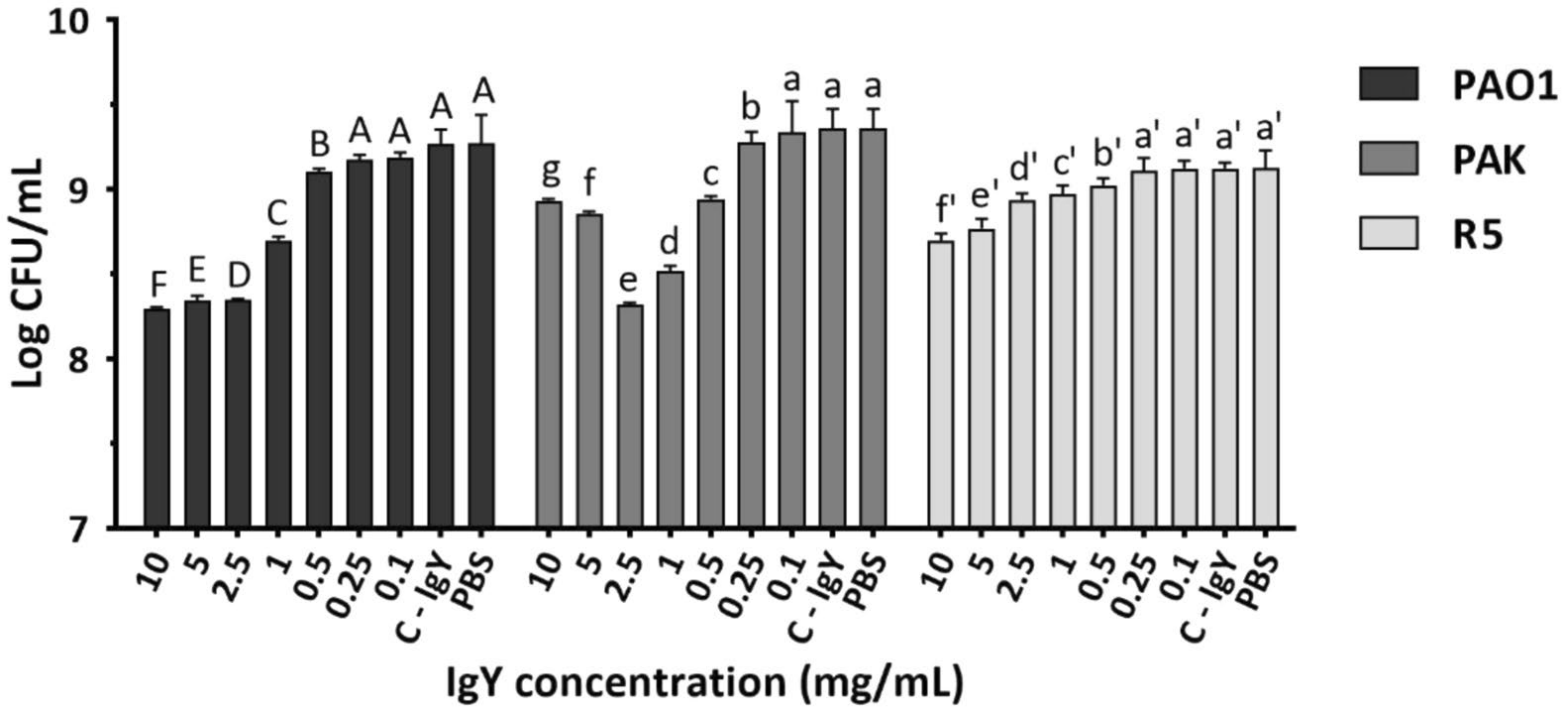
Growth assay results. Growth of *P. aeruginosa* strains in LB broth medium with varying concentrations of specific IgY (ranging from 0.1 to 10 mg/mL), 10 mg/mL of C-IgY (negative control), or ceftazidime at 32 µg/mL (positive control). CFUs were quantified using the spread plate method on LB Agar. Mean comparison of anti-PAO1-IgY at each concentration with control treatments (C-IgY and PBS) for each bacterial strain, in terms of Log CFU/mL, using Duncan’s multiple range test (represented as uppercase, lowercase and primed letters for PAO1, PAK and R5 strains, respectively). For each strain, different letters among each anti-PAO1-IgY dose and control treatments denote significant differences, while sharing at least one letter implies non-significant differences. Values represent the mean of three independent triplicate experiments ± standard error of mean (SEM), PBS; phosphate-buffered saline.

The anti-PAO1-IgY was found to impede the growth of PAO1, PAK and R5 strains with the minimum effective concentrations of 0.5, 0.25 and 0.5 mg/mL, respectively. Conversely, the C-IgY did not display any inhibitory activity against the bacteria (Fig. 2). The inhibitory effect of anti-PAO1-IgY increased in a dose-dependent manner, plateauing at a certain concentration, and entering the hook ranges for PAK strain at 5 mg/mL (Supplementary data, Fig. S2). The anti-PAO1-IgY reduced the growth rates of PAO1, PAK and R5 strains in ranges of 32–89%, 17–91% and 21–63%, respectively.

### The anti-PAO1-IgY decreased the bacterial motility

**Figure 3:**
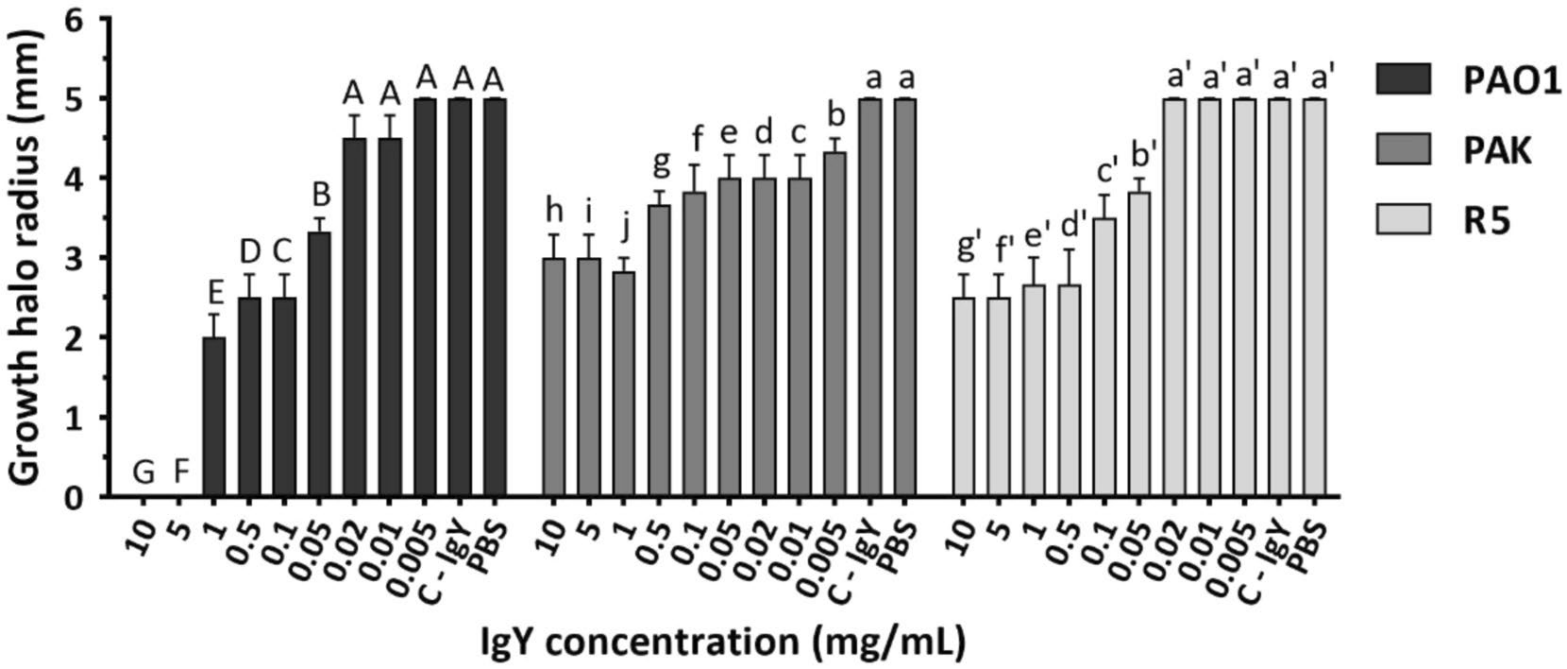
Motility assay results. Pretreated PA strains at varying concentrations of anti-PAO1-IgY were stabbed onto motility agar plates. Plates were incubated at 30 °C for 18 h, and the radii of the growth halos were measured. Mean comparison of anti-PAO1-IgY at each concentration with control treatments (C-IgY and PBS) for each bacterial strain, in terms of growth halo radius (mm), using Duncan’s multiple range test (represented as uppercase, lowercase and primed letters for PAO1, PAK and R5 strains, respectively). For each strain, different letters among each anti-PAO1-IgY dose and control treatments denote significant differences, while sharing at least one letter implies non-significant differences. Values represent the mean of three independent triplicate experiments ± standard error of mean (SEM), PBS; phosphate-buffered saline.

The anti-PAO1-IgY was found to be effective in reducing the motility of PAO1, PAK and R5 strains at concentrations of 0.05, 0.005 and 0.05 mg/mL, respectively, and at higher concentrations (Fig. 3). The inhibitory activity was increased against the homologous strain at higher concentrations, resulting in a 100% potency at 5 mg/mL and reaching the saturation range for the other two strains (Supplementary data, Fig. S3). The anti-PAO1-IgY inhibited the motility of PAO1, PAK and R5 strains in ranges of 23–100%, 20–43% and 23–50%, respectively. The C-IgY was used at the highest examined concentration and showed no inhibitory activity against the bacteria (Fig. 3).

### IgYs increased the cell surface hydrophobicity of bacterial strains

**Figure 4:**
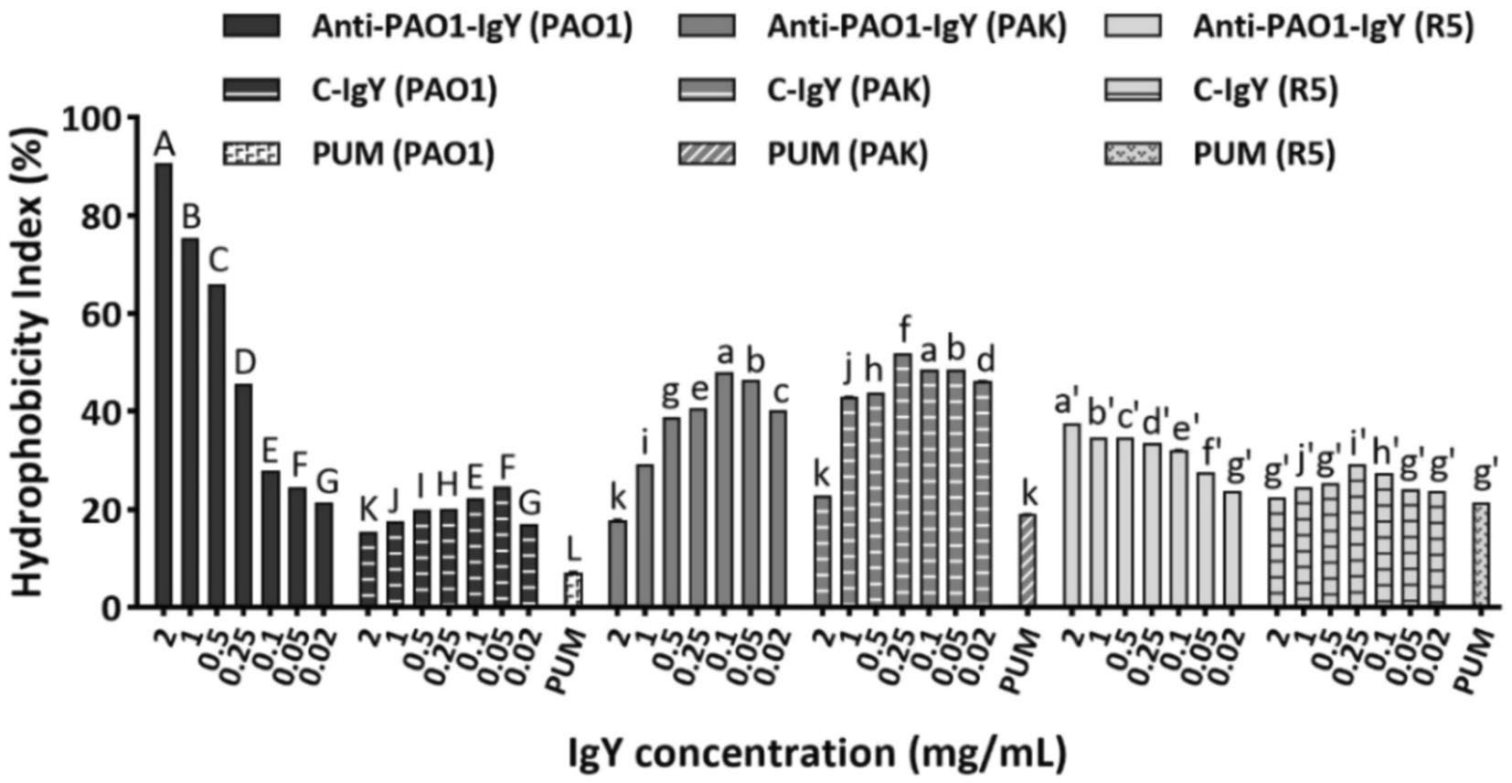
MATH assay results. Aqueous bacterial suspensions were mixed with varying concentrations of anti-PAO1-IgY, and their affinity towards isooctane hydrocarbon was measured through OD readings. Mean comparison of anti-PAO1-, and C-IgYs at each concentration with PUM control for each bacterial strain, in terms of hydrophobicity index (HI) (%), using Duncan’s multiple range test (represented as uppercase, lowercase and primed letters for PAO1, PAK and R5 strains, respectively). For each strain, different letters between anti-PAO1-, and C-IgY groups at each given concentration and PUM control denote significant differences, while sharing at least one letter in common implies non-significant differences. Values represent the mean of two independent triplicate experiments ± standard error of mean (SEM), PUM; phosphate urea magnesium sulfate buffer.

Both anti-PAO1-, and C-IgYs induced significant shifts in absorbance in the MATH assay. The anti-PAO1-IgY conferred the highest changes in CSH of its homologous strain (Fig. 4), and entered the hook values only for the PAK strain, whereas this pattern was observed for all C-IgY-treated strains (Supplementary data, Fig. S4). The anti-PAO1-IgY increased the HI of PAO1, PAK and R5 strains by 3–12.7, 1.5–2.5 and 1.3–1.75 of a fold, respectively. Similarly, the C-IgY conferred the CSH change for the same strains by 2.4–3.4, 2.3–2.7, and 1.1–1.4-fold.

### The anti-PAO1-IgY reduced the biofilm formation ability of bacteria

**Figure 5:**
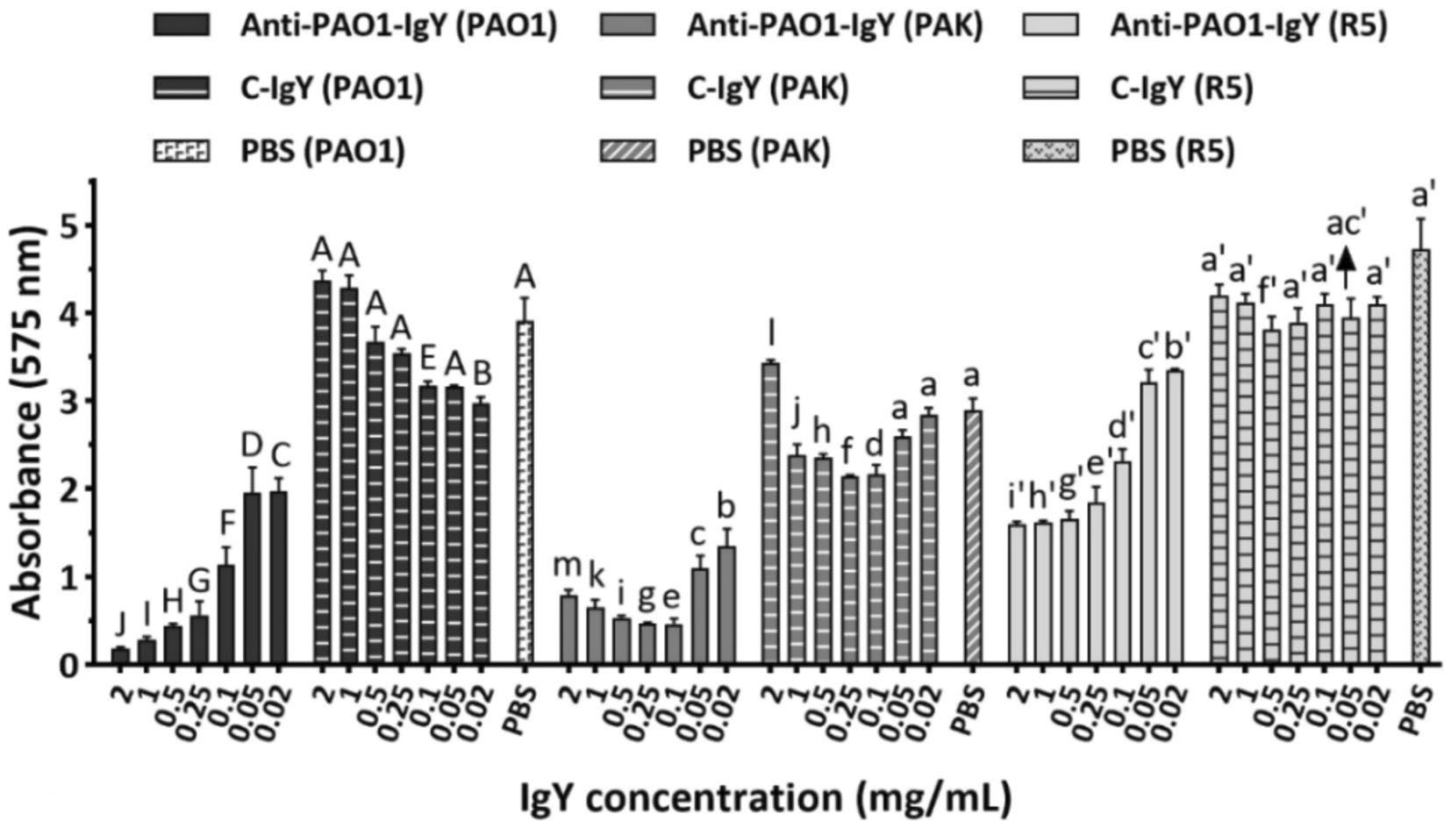
Biofilm assay results. Biofilm formation of bacterial strains incubated at diverse IgY concentrations in microtiter plates visualized through crystal violet staining. Mean comparison of anti-PAO1-, and C-IgYs at each concentration with PBS control for each bacterial strain, in terms of absorbance (575 nm), using Duncan’s multiple range test (represented as uppercase, lowercase and primed letters for PAO1, PAK and R5 strains, respectively). For each strain, different letters between anti-PAO1-, and C-IgY groups at each given concentration and PBS control denote significant differences, while sharing at least one letter in common implies non-significant differences. Values represent the mean of two independent sextuplicate experiments ± standard error of mean (SEM), PBS; phosphate-buffered saline.

The anti-PAO1-IgY effectively decreased the biofilm formation ability of bacterial strains in all examined concentrations and the degree of inhibition varied proportionally with the dosage (Fig. 5). The inhibitory effect entered the saturation range at 0.25 and 0.1 mg/mL for PAO1/R5 and PAK strains, respectively (Supplementary data, Fig. S5). The anti-PAO1-IgY inhibited the biofilm formation of the PAO1, PAK and R5 strains by 50–95%, 54–84% and 29–66%, respectively. Also, the C-IgY conferred a slight reduction in biofilm formation in some of the examined concentrations (Fig. 5).

### The anti-PAO1-IgY reduced the internalization efficacy of bacterial strains

**Figure 6:**
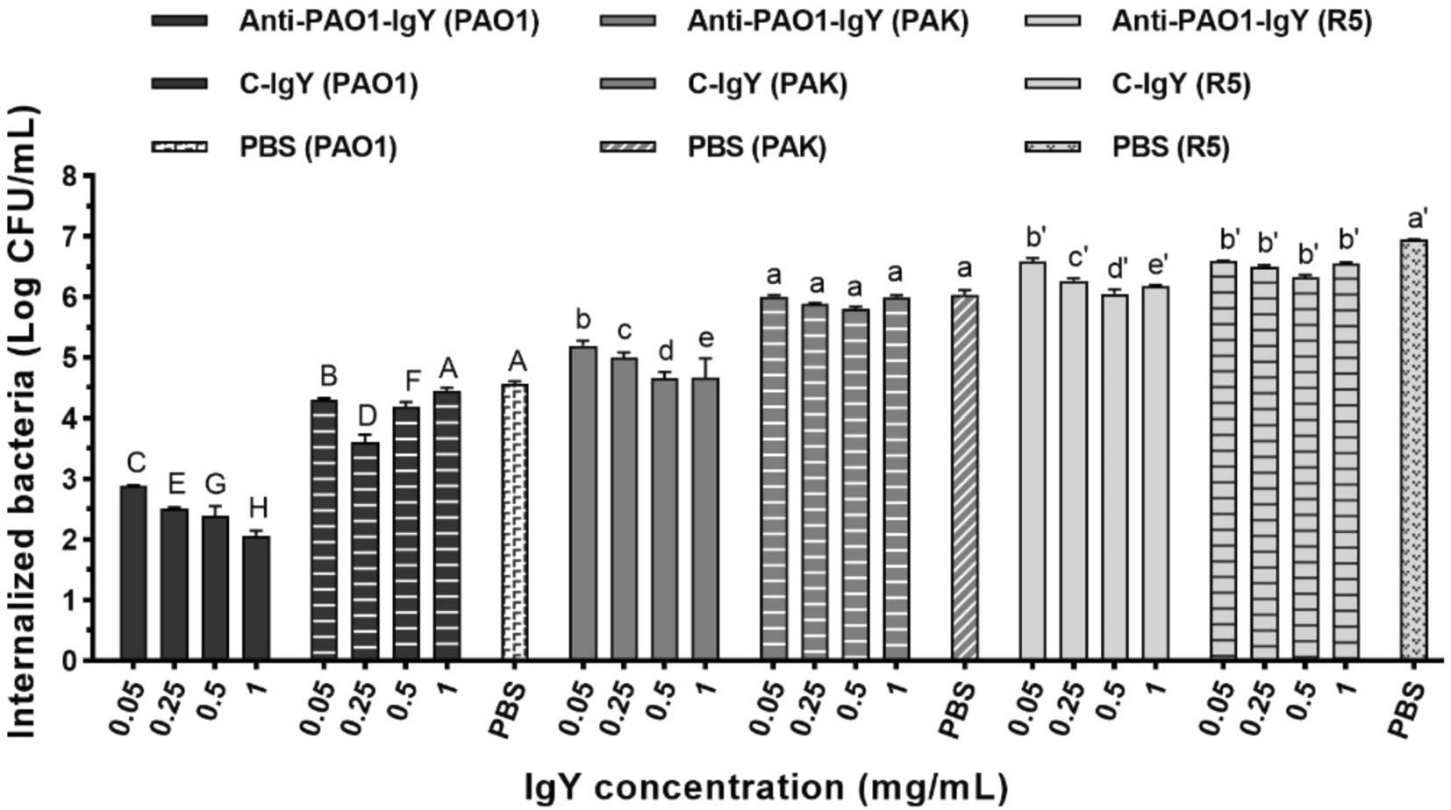
Cell internalization assay results. Monolayers of A549 cells were subjected to a challenge with 2 × 10^8^ CFUs/well of pretreated PA strains at varying anti-PAO1-IgY concentrations. The internalized bacteria were subsequently released, serially diluted and plated onto LB agar. Mean comparison of anti-PAO1-, and C-IgYs at each concentration with PBS control for each bacterial strain, in terms of internalized bacteria (Log CFU/mL), using Duncan’s multiple range test (represented as uppercase, lowercase and primed letters for PAO1, PAK and R5 strains, respectively). For each strain, different letters between anti-PAO1-, and C-IgY groups at each given concentration and PBS control denote significant differences, while sharing at least one letter in common implies non-significant differences. Values represent the mean of two independent triplicate experiments ± standard error of mean (SEM), *PBS* phosphate-buffered saline.

The anti-PAO1-IgY showed inhibitory efficacy against the cell internalization ability of bacteria at all examined concentrations. The antibody showed the highest efficacy against the PAO1, PAK and R5 strains, respectively (Fig. 6). It inhibited the internalization of PAO1, PAK and R5 strains by 37–55%, 14–23% and 5–13%, respectively. However, the C-IgY showed no inhibitory effect against the PAK and R5 strains. Still, it exhibited a slight effect against the PAO1 strain at 0.05–0.5 mg/mL (Fig. 6), with a hook effect observed at 0.5 mg/mL (Supplementary data, Fig. S6).

### The anti-PAO1-IgY boosted the opsonophagocytic activity of polymorphonuclear leukocytes

**Figure 7:**
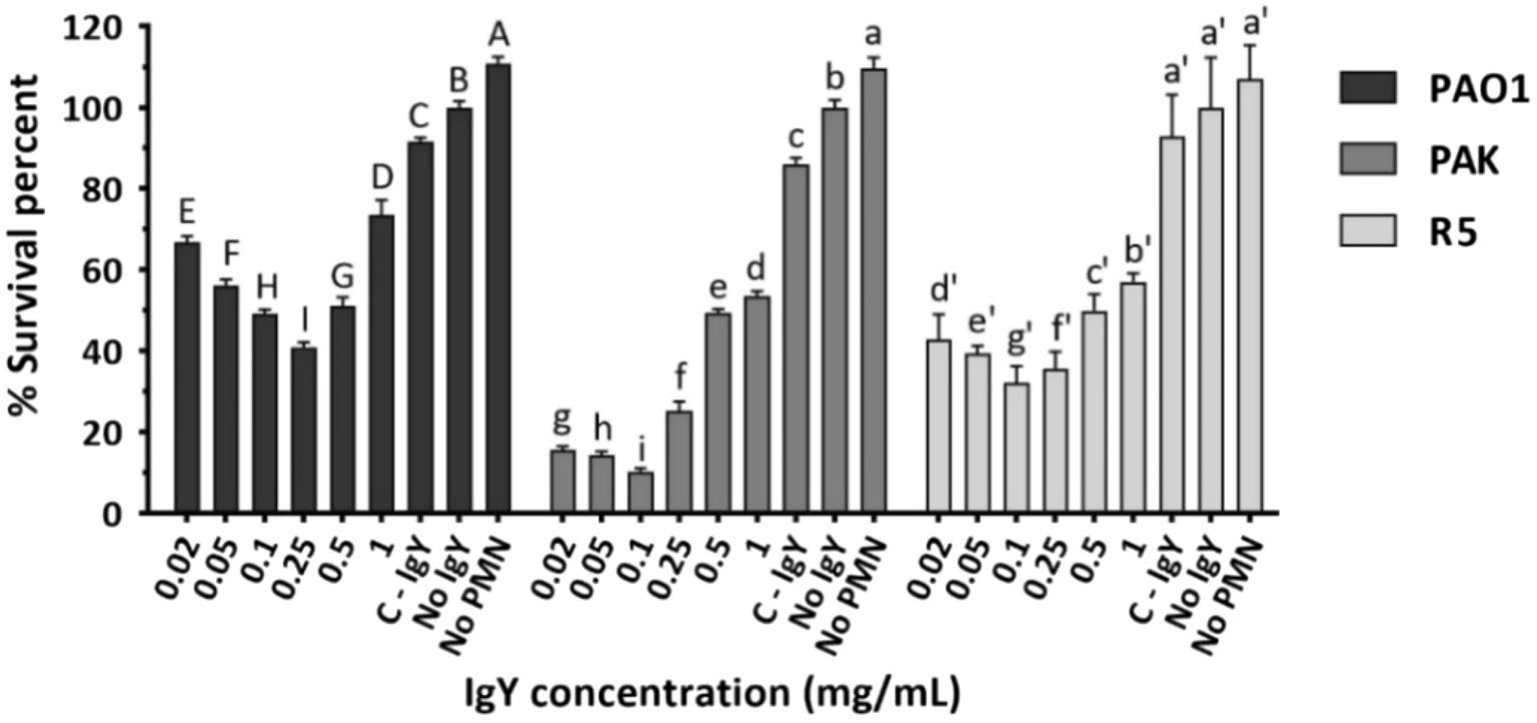
Opsonophagocytic assay results. Bacterial strains, pretreated at varying concentrations of anti-PAO1-IgY, were incubated with 10^5^ neutrophils. The surviving bacteria were then plated on LB agar. Mean comparison of anti-PAO1-IgY at each concentration with control treatments (C-IgY, No IgY, and No-PMN) for each bacterial strain, using Duncan’s multiple range test (represented as uppercase, lowercase and primed letters for PAO1, PAK and R5 strains, respectively). For each strain, different letters among each anti-PAO1-IgY dose and control treatments denote significant differences, while sharing at least one letter in common implies non-significant differences. Values represent the mean of two independent experiments carried out in triplicate ± standard error of mean (SEM), *PMN* polymorphonuclear leukocytes.

The anti-PAO1-IgY augmented the PMN-mediated opsonophagocytic activity in a dose-dependent manner. The lowest survival rates for the PAO1 and PAK/R5 strains were observed at concentrations of 0.25 and 0.1 mg/mL, respectively (Fig. 7). The survival deduction efficacy of the antibody was reduced upon dose increase due to hook effect at higher concentrations (Supplementary data, Fig. S7). Compared to the No-IgY control survival, the anti-PAO1-IgY was observed to decrease the survival percentage of the PAO1, PAK and R5 strains by 26–59%, 46–90% and 43–68%, respectively. The C-IgY also reduced the survival rate of the PAO1 and PAK strains by approximately 8% and 14%, respectively (Fig. 7). However, it showed no efficacy against the hospital strain.

### The anti-PAO1-IgY efficaciously protected the infected burned mice and decreased the bacterial burden

**Figure 8:**
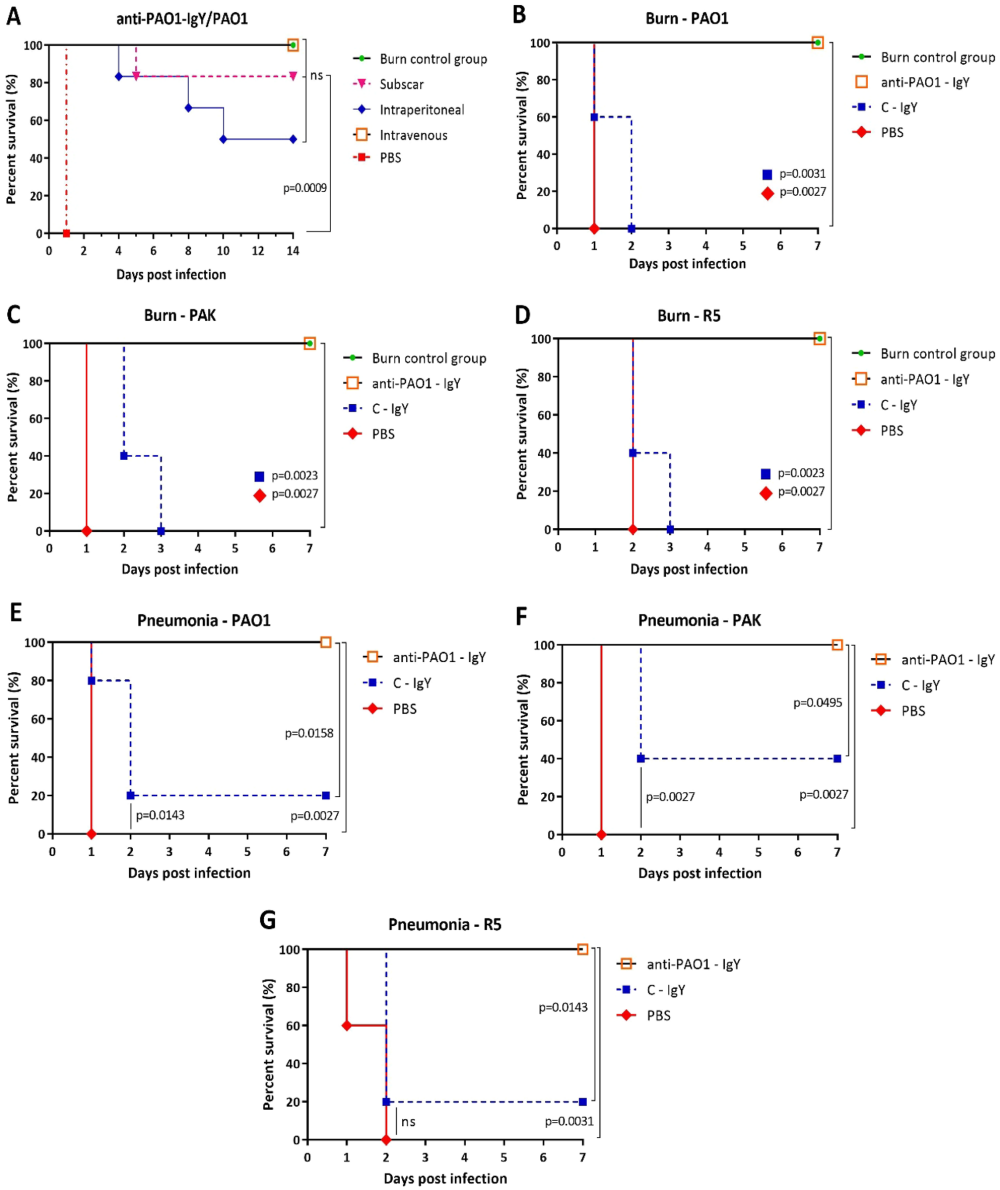
Survival results. Passive immunotherapy effects of anti-PAO1-IgY via different administration routes on PAO1-infected burned mice (n = 6) **(A)**, and the protective efficacy of avian antibodies on the survival of mice (n = 5) infected with *P. aeruginosa* PAO1 **(B,E)**, PAK **(C,F)**, and R5 **(D,G)**, in burn wound **(B–D)** and acute pneumonia **(E–G)** models. Survival rates were monitored for one week following infection. P-values were calculated using the Mantel–Cox log-rank test, *PBS* phosphate-buffered saline.

**Figure 9:**
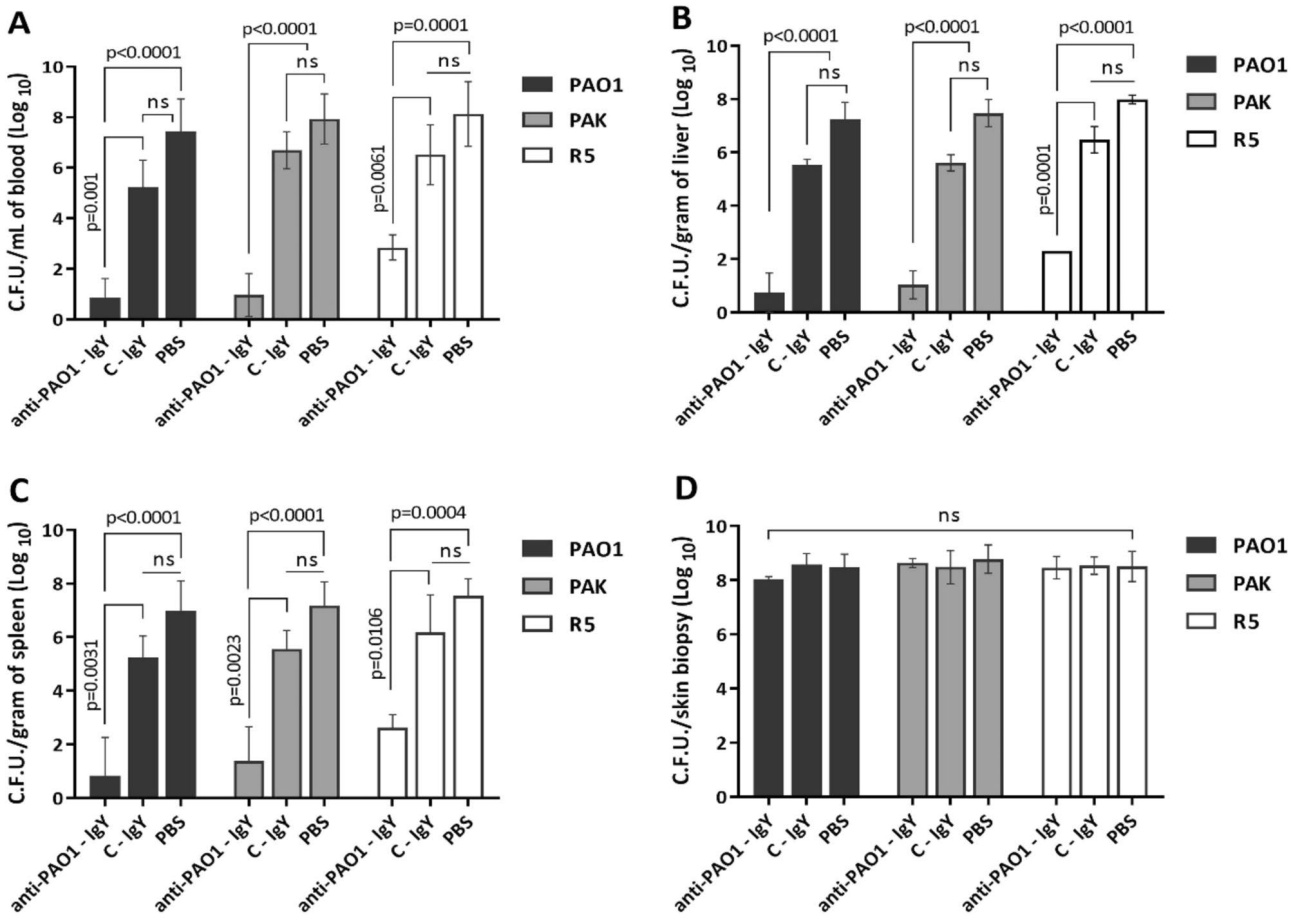
Bacterial load examination. The anti-PAO1-IgY efficacy in reducing the bacterial burden in the blood **(A)**, liver **(B)**, spleen **(C)** and skin biopsies **(D)** of burned mice infected with bacterial strains. Values represent the mean of triplicate independent experiments ± standard error of mean (SEM). The P-values were calculated using Tukey's multiple comparisons test, *ns* not significant, *PBS* phosphate-buffered saline.

Based on preliminary animal experiments, the lethal doses of the PAO1, PAK and R5 strains were determined to be 10^8^, 4 × 10^8^ and 10^8^ CFUs, respectively. The findings also revealed that a 1 mg dosage of IgY represented the lowest amount required to achieve a superior survival rate (Supplementary data, Fig S8). Passive immunotherapy with anti-PAO1-IgY provided 100, 83 and 50% protection in PAO1-infected burned mice via intravenous, subscar and intraperitoneal administration routes, respectively (Fig. 8A). The anti-PAO1-IgY yielded 100% protection against all bacterial strains in burn wound murine model. The C-IgY only delayed the death of a few mice and did not offer protection against infection. All mice in the non-infected burn control group survived for up to 9 weeks (Fig. 8B–D). Bacterial burden examinations confirmed the observed survival rates. Passive immunotherapy with anti-PAO1-IgY significantly reduced the bacterial loads in blood, liver and spleen of infected burned mice in comparison to C-IgY and PBS groups (Fig. 9A–C). The anti-PAO1-IgY decreased the blood-liver-spleen bacterial loads of PAO1, PAK and R5-infected mice by 88–90–88%, 88–86–81% and 65–72–68%, respectively. On the other hand, the C-IgY showed a reduction in the blood bacterial load of PAO1-infected mice and the liver bacterial loads of PAK and R5-infected mice by 29%, 24%, and 18%, respectively. However, no significant difference was observed between C-IgY and PBS groups. Furthermore, no significant difference was observed in the bacterial loads of the skin biopsies among the test, control, and PBS mice groups (Fig. 9D).

### The anti-PAO1-IgY effectively protected mice against lethal PA pneumonia

The anti-PAO1-IgY provided 100% protection against all bacterial strains in acute pneumonia murine model. Also, the C-IgY conferred 20% and 40% protection in PAO1/R5-, and PAK-infected mice, respectively (Fig. 8E–G).

## Discussion

The emergence of highly virulent and MDR clinical PA isolates presents serious healthcare challenges primarily due to their rapid dissemination throughout hospitals worldwide along with the concern of a decreasing number of effective antibacterial agents^28–32^. In addition, clinical PA isolates are known to produce numerous virulence factors that play a critical role in biofilm formation, leading to chronic infections owing to inefficient antimicrobial therapy^33–35^. Fortunately, growing evidence indicates that IgY immunotherapy has great therapeutic potential as a stand-alone treatment method or in conjunction with antibiotics against microbial infections^36–46^. Inactivated whole-cell bacterial cells hold the most promise among PA antigens due to containing a variety of protective antigens that enable clinical PA strains to colonize and invade host tissues^47–49^. Our recent studies have shown the efficacy of IgY immunotherapy against PcrV, OprF, and flagellin proteins, in murine models of PA infection^43,44,46^. Here, we present the effectiveness of anti-PAO1-IgYs in the treatment and control of potentially fatal PA infections. Our findings demonstrated that mice were completely protected against infection caused by heterologous PA strains, and their survival rates were considerably higher than those of mice previously passively immunized with IgY against flagellin^46^. Indeed, mentioning notable survival rates of passively immunized mice that were challenged with heterologous strains, immunotherapy with avian anti-PAO1 antibodies’ efficacy surpasses our former mono- or bivalent series of vaccines and immunotherapy approaches against PA^43,44,46,50–53^. Moreover, the finding that anti-PAO1-IgYs cross-react with all PA strains indicates that this immunotherapy strategy has the potential to contribute effective protection against infections caused by various clinical PA isolates. The notion of anti-PA-IgY is a promising efficacious prophylactic treatment that interferes with multiple antigens that mirror many of those presented by nosocomial fully virulent PA strains^54–56^.

Our assessment of in vitro opsonophagocytic and motility inhibition assays have indicated that anti-PAO1-IgYs have robust opsonic killing and immobilizing activities against all PA strains. Recent studies have revealed that the high hydrophobicity of anti-PA-IgYs can causes physical modification, resulting in immobility and formation of PA aggregates, consequently, supporting the phagocytic clearance by neutrophils^57^. Similarly, Aztreonam has been shown to increase bacterial cell surface hydrophobicity, which promotes bacterial interaction with leukocyte membranes, resulting in phagocytic engulfment^58^. Moreover, the high hydrophobicity of IgY, compared to mammalian IgG, allows the Fc component to be orientated opposing the face of the antigen, revealing it to the Fc receptor, which causes efficient internalization of PA strains by neutrophils^57^. In vitro studies have also demonstrated that anti-PA-IgYs are capable of enhancing the production of reactive oxygen species (ROS) during the PMN-mediated phagocytosis and killing of PA^59^. The weak anti-PA activity of IgY from non-immunized hens may be attributed to the chickens' exposure to PA, which is a ubiquitous environmental bacterium. As a result, their immune systems may produce some natural antibodies against the bacteria, but these antibodies are not effective enough to provide full protection against PA infections. Remarkably, anti-PA-IgYs have shown potential for inhibiting biofilm formation, which is a major obstacle in the management of PA infections due to the bacteria's intrinsic resistance to many antibiotics and biocides. This suggests that anti-PA IgY immunotherapy may offer a new approach to the prevention and treatment of biofilm-associated PA infections^60^. Also, anti-PA IgYs have been found to inhibit bacterial adherence to epithelial cells and augment mucosal IgA immunity. These effects may be beneficial in preventing PA colonization, which is a crucial initial step in the onset of PA infection. By inhibiting bacterial adherence, anti-PA-IgYs may reduce the risk of infection and enhance the body's ability to clear PA from mucosal surfaces. These findings suggest that anti-PA-IgY immunotherapy may have potential for preventing and treating PA infections, particularly those involving mucosal surfaces^25,26^. This implies that anti-PA-IgYs suppress the key virulence factors of PA which are responsible for promoting bacterial colonization, biofilm formation, internalization, and eventual systemic dissemination in murine models, leading to greater survival rates in passively immunized animals in comparison to control group^61^. In contrast to the previously described low anti-motility and opsonic-killing activities of anti-FlaA-IgY against a heterologous PA strain, anti-PA-IgYs cross-react with all PA strains, indicating that the antibodies have high protective activity against heterologous strains^46^. Interestingly, the protective effect of anti-PA-IgYs outperforms that of IgYs generated against flagellin, PcrV, and OprF, indicating that anti-PA-IgYs have broad-spectrum activity against all PA strains^43,44,46^. Taken together, these results show that anti-PAO1-IgYs can protect mice against PA infections, probably by inducing opsonophagocytosis and inhibiting bacterial motility, biofilm formation, and internalization^57,62–64^.

Data from the potency evaluation of anti-PA-IgYs demonstrated that the immunotherapy approach effectively inhibited the systemic spread of PA in infected mice, as evidenced by a significant decrease in the bacterial loads of the blood, spleen, and liver. Unlike IgYs generated against mono- and bivalent antigens, passive immunization with anti-PA-IgYs thwarts the systemic dissemination of all PA strains from the site of infection to the liver^43,44,46^. These data support the notion that immunotherapy with anti-PA-IgYs decreases bacterial burden by disrupting the early stages of PA pathogenesis, prior to colonization in the liver^59,65,66^. Immunotherapy with avian anti-PA antibodies offers overall benefits over polyclonal and monoclonal IgG in the treatment of PA infections^66,67^. IgY-treated lung epithelial cells had considerably lower levels of pro-inflammatory cytokines than goat and human IgG, showing that IgYs do not cause inflammatory responses and are safe for use in preventing airway infections^66,67^. Likewise, the level of pro-inflammatory cytokines in the serum of PA-infected IgY-treated mice was significantly lower than that of the control group, demonstrating modulation of neutropoiesis in response to decreasing bacterial numbers by opsonophagocytosis^66,67^. Early binding of IgYs to PA could inhibit bacterial growth and colonization of the mucosal surface by reducing bacterial adherence to the oropharynx^62,66^. Studies have shown that oral IgY immunotherapy against rotavirus and pulmonary PA infections has been safe with no reported no side effects for up to ten years^62,66,68^. As a result, passive immunization of cystic fibrosis (CF) patients with IgY can enhance mucosal IgA immunity, which in turn prevents pulmonary colonization by PA^62,69^**.** It is worth noting that IgY in chicken eggs is generally well tolerated and is considered as an essential component of a healthy human diet. Gargling with an anti-pseudomonal IgY in CF patients every night after brushing teeth helped to reduce the incidence of PA infections in CF patients, likely by preventing the initial colonization of the bacteria in the oropharynx^15,70^. Furthermore, in a mechanically ventilated porcine model, administration of anti-PA-IgYs led to a reduction in the growth and colonization of PA^56^. In CF patients, oral IgY treatment in conjunction with clarithromycin protected PA colonization and lung infection, resulting in delayed or avoided chronic infection^71,72^. As indicated by the above-mentioned findings, IgYs have great potential in the prevention and treatment of PA infections due to their high target specificity, great binding avidity, and lack of cross-reactivity with mammalian Fc receptors^73–76^.

Finally, we evaluated whether immunotherapy with anti-PAO1-IgYs could improve the survival of PA-infected mice. Many studies in the realm of IgY therapeutic applications have consistently shown that while these antibodies have been successful in inhibiting the initial colonization of bacteria, they have proven ineffective in preventing systemic infections^56,77^. A recently published report by Zamani et al. delved into the efficacy of passive immunotherapy using IgY antibodies raised against the chimeric protein pilQ-pilA-DSL region. Their findings revealed that despite exhibiting appropriate reactivity and a partial reduction in inflammatory mediators, these antibodies were unable to protect rabbits from *P. aeruginosa* sepsis^77^. In light of these challenges, we carefully timed the administration of antibodies to thwart bacterial colonization from the very first hours of exposure. We also administered subsequent doses to target any bacteria that might have invaded deeper tissues, thus aiming to prevent systemic infection and mortality. Since the survival assay yielded the most favorable results, we did not delve into further examination of alternative regimens. We showed that compared to previously described IgY immunotherapy against flagellin, PcrV, and OprF, immunotherapy with anti-PA IgYs provides broader spectrum protection against PA infection caused by heterologous strains^43,44,46^. The survival rates show that following infection, passively immunized mice become resistant to infection caused by heterologous strains, eventually clearing PA infections^59,65,66^. Anti-PA-IgYs appear to act against the PA virulence factors which are crucial in PA colonization and invasion in the early phases of PA infection^78–80^. The findings demonstrating a considerable increase in survival of passively immunized mice align with the heightened in vitro protective activities of IgY observed in this study, including opsonophagocytic, anti-biofilm, and anti-motility activities, accompanied by a considerable reduction in liver bacterial loads^78–80^. We declare that our findings are limited to evaluating the protective efficacy of immunotherapy using anti-PA-IgYs in mice. Nonetheless, immunotherapy with these antibodies has the potential to provide broad-spectrum protection against various PA infections, owing to its multivalency and propensity to interfere with heterologous strains. Our findings warrant a thorough study to address the aforementioned issues and to test the efficacy of passive immunization with anti-PA-IgYs in various models of PA infections.

### Supplementary Information

Supplementary Figures.

## Data Availability

The datasets generated and/or analyzed during the current study are available from the corresponding author on reasonable request.
