# Supplementary material for: Polyclonal anti-whole cell IgY passive immunotherapy shields against P. aeruginosa-induced acute pneumonia and burn wound infections in murine models
Source: Sci Rep. 2024 Jan 3;14:405. doi: 10.1038/s41598-023-50859-x (PMC10764880; doi:10.1038/s41598-023-50859-x)

**Fig. S1.** **Purity and immunoreactivity of anti-PAO1-IgY;** (**A);** SDS-PAGE analysis of produced IgY antibodies, separated on 9% (w/v) SDS-polyacrylamide gel, revealing two main bands corresponding to light chains (∼25 kDa) and heavy chain (∼65 kDa) of the immunoglobulins, M; SMOBIO PM2600 10-245 kDa protein weight marker, 1: anti-PAO1-IgY, 2: C-IgY, X: unrelated samples. **(B and C);** Mean comparison of each IgY group reactivity against PAO1 **(B)** and PAK **(C)** strains at different time points, using Duncan’s multiple range test, represented as uppercase and lowercase letters for anti-PAO1-, and C-IgYs, respectively. For each IgY, different letters denote significant differences, while sharing at least one letter in common implies non-significant differences. Arrows indicate the immunization weeks (0, 2 and 5). Values represent the mean of three independent triplicate experiments ± standard error of mean (SEM).


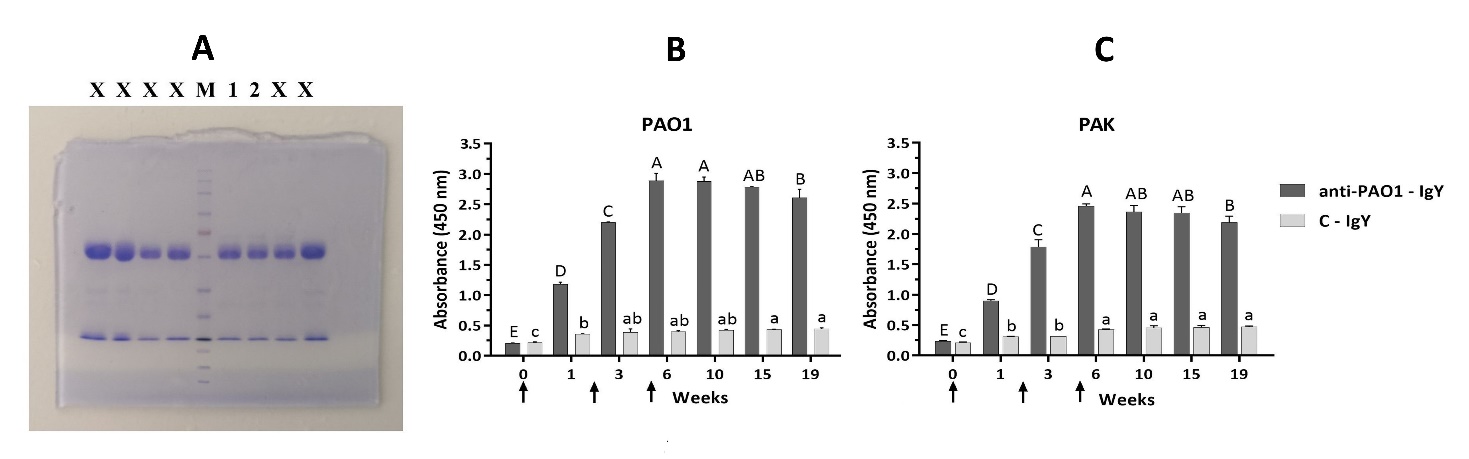


**Fig. S2.** **Growth assay results.** Mean comparison of each bacterial strain growth at varying concentrations of anti-PAO1-IgY in terms of Log CFU/mL using Duncan’s multiple range test, represented as uppercase, lowercase and primed letters for PAO1, PAK and R5 strains, respectively. For each strain, different letters among columns denote significant differences, while sharing at least one letter in common implies non-significant differences. Values represent the mean of three independent triplicate experiments ± standard error of mean (SEM).


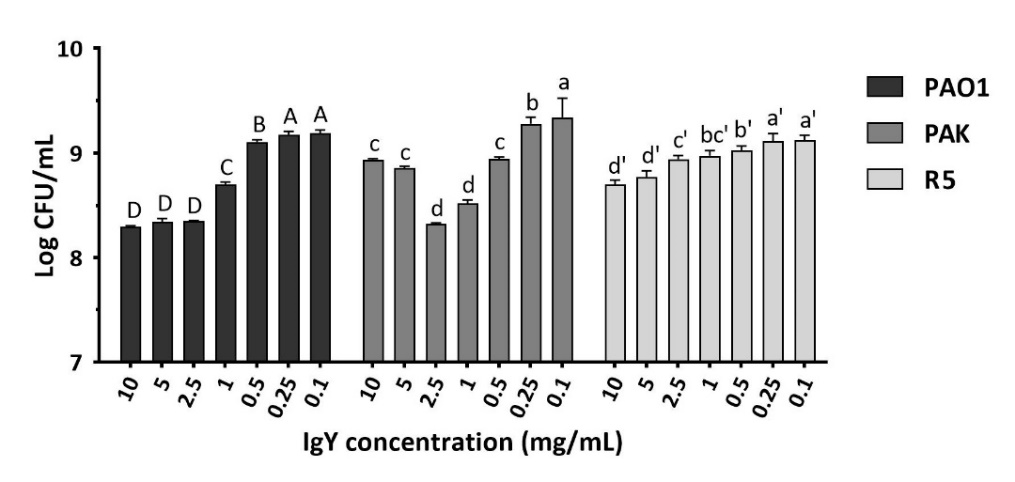


**Fig. S3. Motility assay results.** Mean comparison of each bacterial strain motility at varying concentrations of anti-PAO1-IgY in terms of growth inhibition radius (mm) using Duncan’s multiple range test, represented as uppercase, lowercase and primed letters for PAO1, PAK and R5 strains, respectively. For each strain, different letters among columns denote significant differences, while sharing at least one letter in common implies non-significant differences. Values represent the mean of three independent triplicate experiments ± standard error of mean (SEM).


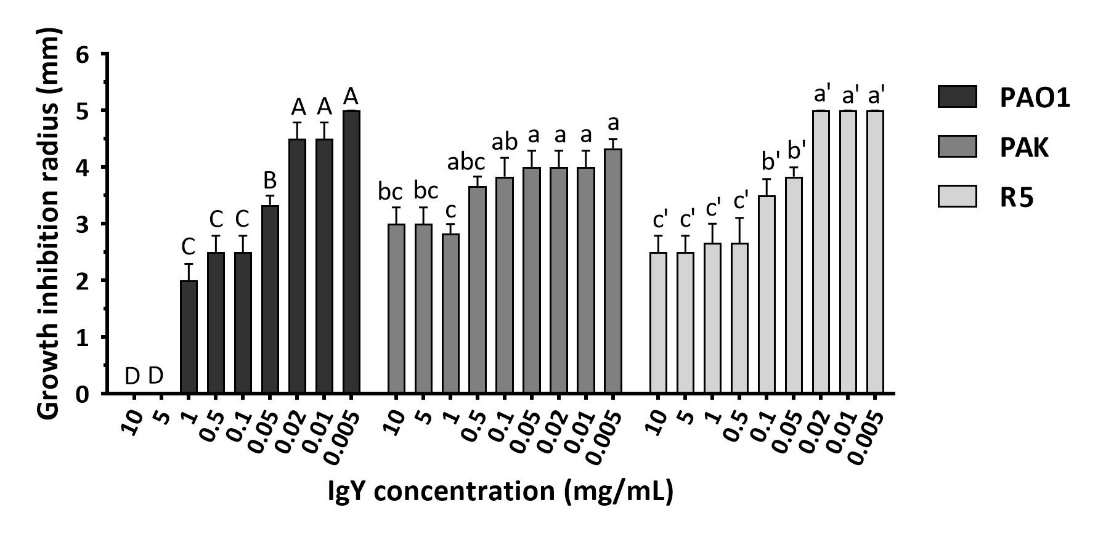


**Fig. S4. MATH assay results.** Mean comparison of each bacterial strain CSH, individually at varying concentrations of anti-PAO1-, and C-IgY in terms of hydrophobicity index (HI) (%) using Duncan’s multiple range test, represented as uppercase, lowercase and primed letters for PAO1, PAK and R5 strains, respectively. For each strain, different letters among columns for each IgY group denote significant differences, while sharing at least one letter in common implies non-significant differences. Values represent the mean of two independent triplicate experiments ± standard error of mean (SEM).


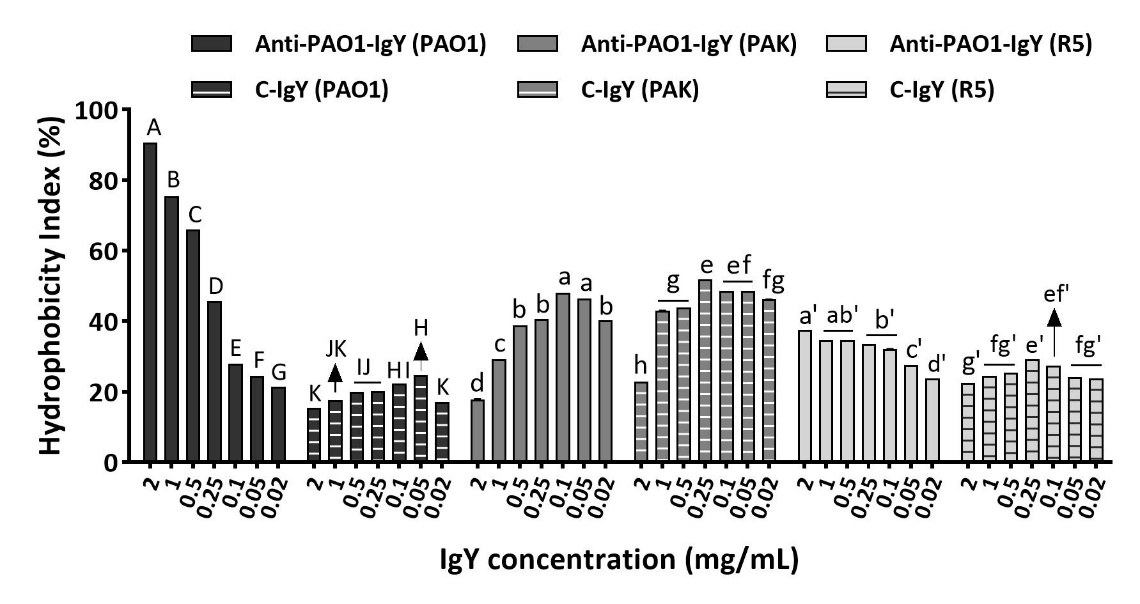


**Fig. S5. Biofilm assay results.** Mean comparison of each bacterial strain biofilm formation ability, individually at varying concentrations of anti-PAO1-, and C-IgY in terms of absorbance (575 nm) using Duncan’s multiple range test, represented as uppercase, lowercase and primed letters for PAO1, PAK and R5 strains, respectively. For each strain, different letters among columns for each IgY group denote significant differences, while sharing at least one letter in common implies non-significant differences. Values represent the mean of two independent sextuplicate experiments ± standard error of mean (SEM).


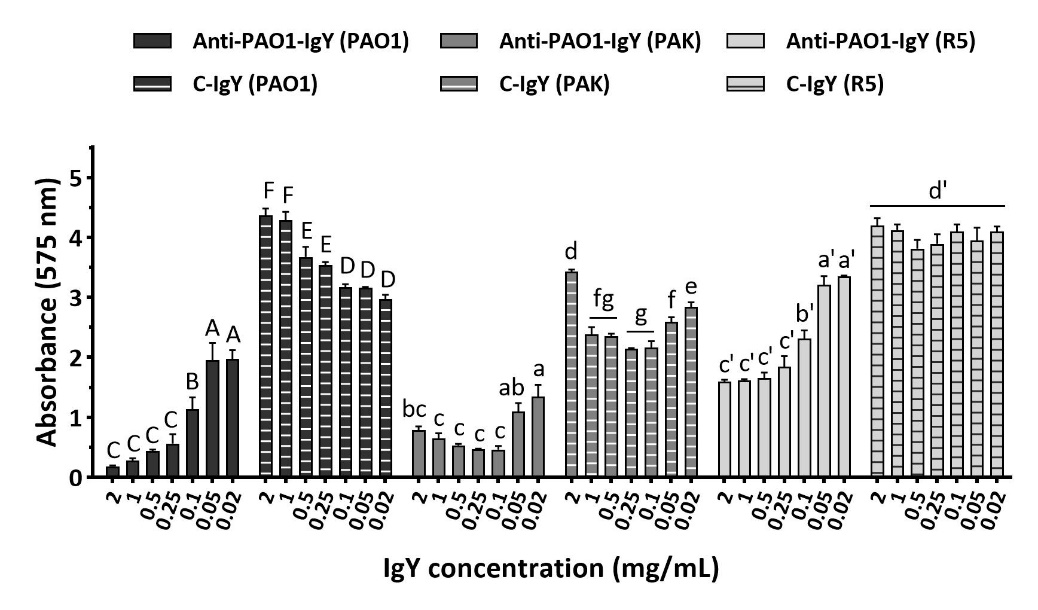


**Fig. S6. Cell invasion assay results.** Mean comparison of each bacterial strain invasion ability, individually at varying concentrations of anti-PAO1-, and C-IgY in terms of invading bacteria (Log CFU/mL) using Duncan’s multiple range test, represented as uppercase, lowercase and primed letters for PAO1, PAK and R5 strains, respectively. For each strain, different letters among columns for each IgY group denote significant differences, while sharing at least one letter in common implies non-significant differences. Values represent the mean of two independent triplicate experiments ± standard error of mean (SEM).


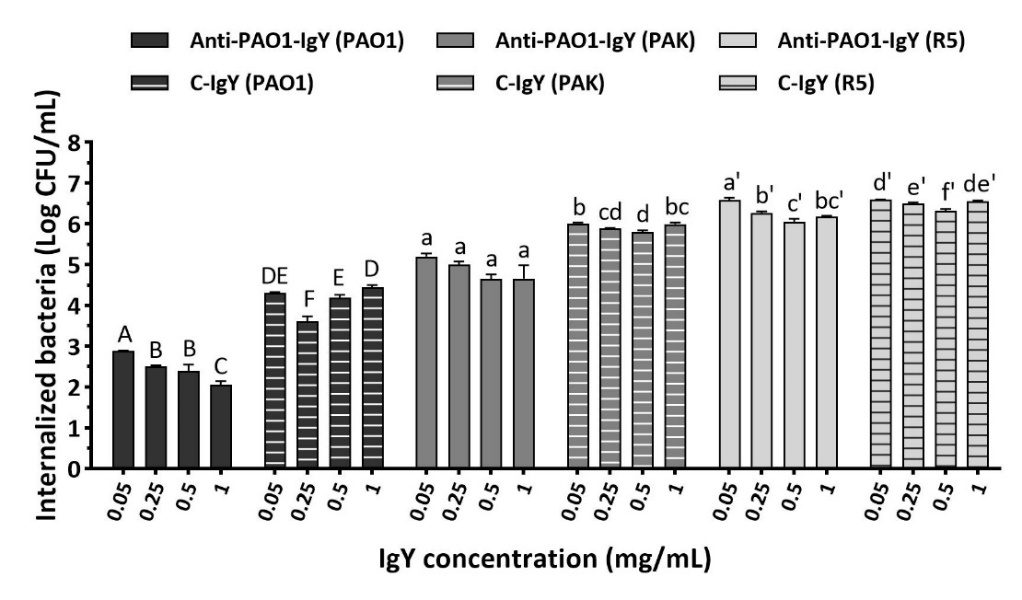


**Fig. S7. Opsonophagocytic assay results.** Mean comparison of each bacterial strain survival between different concentrations of anti-PAO1-IgY in terms of survival percent (%) using Duncan’s multiple range test, represented as uppercase, lowercase and primed letters for PAO1, PAK and R5 strains, respectively. For each strain, different letters among columns denote significant differences, while sharing at least one letter in common implies non-significant differences. Values represent the mean of two independent triplicate experiments ± standard error of mean (SEM).


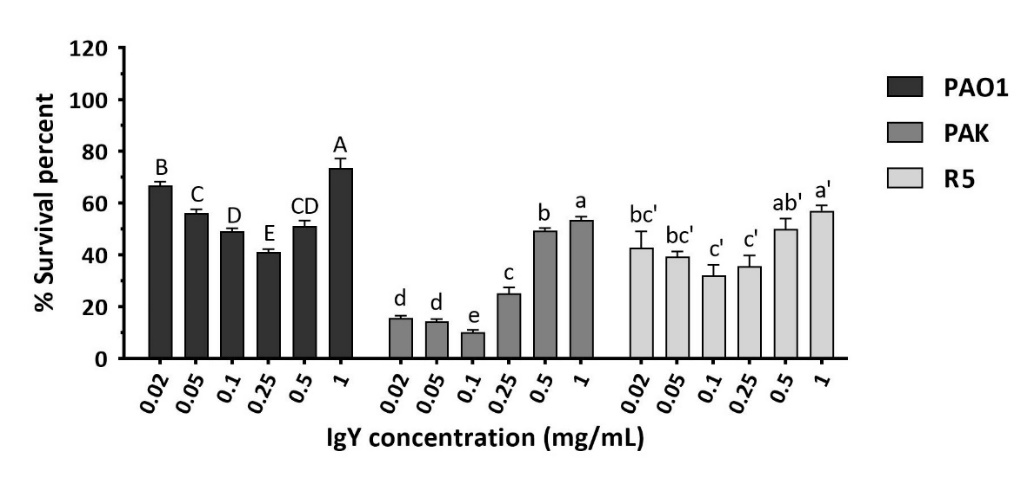


**Fig. S8.** **Primary survival assays.** The survival results of the pilot studies conducted to ascertain the optimal IgY dosage (**A**, n=6) and the lethal doses of PAO1 **(B)**, PAK **(C)** and R5 **(D)** strains (n=3). Four different IgY dosages (0.5, 1, 1.5 and 2 mg) were selected to be examined in the pilot study. BALB/c mice underwent passive immunization with different IgY doses and infected with *P. aeruginosa* PAO1 strain. The lethal dose of *P. aeruginosa* in mice was determined by inoculating mice with varying numbers of bacterial strains. After infection, the mice were maintained under standard laboratory conditions with free access to food and water. The mortality rates were daily monitored for 4 days post infection.


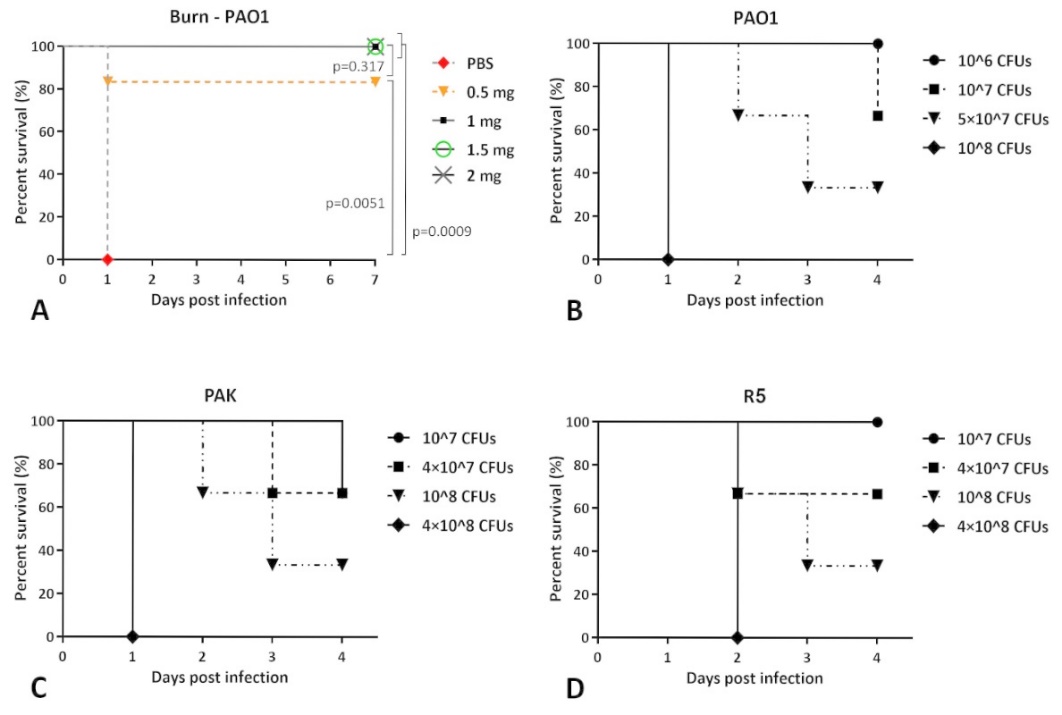

Supplement: Supplementary file 1 — Supplementary Figures. [file 41598_2023_50859_MOESM1_ESM.docx]
